# What Guidance Are Researchers Given on How to Present Network Meta-Analyses to End-Users such as Policymakers and Clinicians? A Systematic Review

**DOI:** 10.1371/journal.pone.0113277

**Published:** 2014-12-17

**Authors:** Shannon M. Sullivan, Doug Coyle, George Wells

**Affiliations:** 1 University of Ottawa Heart Institute, Ottawa, Ontario, Canada; 2 University of Ottawa, Department of Epidemiology and Community Medicine, Ottawa, Ontario, Canada; National Taiwan University, Taiwan

## Abstract

**Introduction:**

Network meta-analyses (NMAs) are complex methodological approaches that may be challenging for non-technical end-users, such as policymakers and clinicians, to understand. Consideration should be given to identifying optimal approaches to presenting NMAs that help clarify analyses. It is unclear what guidance researchers currently have on how to present and tailor NMAs to different end-users.

**Methods:**

A systematic review of NMA guidelines was conducted to identify guidance on how to present NMAs. Electronic databases and supplementary sources were searched for NMA guidelines. Presentation format details related to sample formats, target audiences, data sources, analysis methods and results were extracted and frequencies tabulated. Guideline quality was assessed following criteria developed for clinical practice guidelines.

**Results:**

Seven guidelines were included. Current guidelines focus on how to conduct NMAs but provide limited guidance to researchers on how to best present analyses to different end-users. None of the guidelines provided reporting templates. Few guidelines provided advice on tailoring presentations to different end-users, such as policymakers. Available guidance on presentation formats focused on evidence networks, characteristics of individual trials, comparisons between direct and indirect estimates and assumptions of heterogeneity and/or inconsistency. Some guidelines also provided examples of figures and tables that could be used to present information.

**Conclusions:**

Limited guidance exists for researchers on how best to present NMAs in an accessible format, especially for non-technical end-users such as policymakers and clinicians. NMA guidelines may require further integration with end-users' needs, when NMAs are used to support healthcare policy and practice decisions. Developing presentation formats that enhance understanding and accessibility of NMAs could also enhance the transparency and legitimacy of decisions informed by NMAs.

## Introduction

Transparency is a key principle underlying fair and legitimate health technology assessment (HTA) processes and related policy decisions [1]. Transparency requires not only providing sufficient information, but also providing information in an accessible and understandable format for end-users. This is especially relevant when complex methods such as network meta-analyses (NMA) form the basis of a HTA.

In the absence of head-to-head trials of relevant comparators, NMAs frequently inform cost-effectiveness evaluations and therapeutic or drug class reviews [2]. One frequently cited concern with NMAs is that the complex statistical methods do not permit the end user to understand how the results were obtained or if they are valid [3], [4]. Creating simple but accurate explanations of NMAs for policymakers, and those impacted by policy decisions such as clinicians and patients can be challenging. Currently, this is further complicated by the variable expertise among researchers in conducting and interpreting NMAs [3], [5], the rapidly evolving developments in NMA methods and restrictions inherent in different methodological approaches (e.g. Bayesian versus frequentist analyses) and available NMA software (e.g. WinBugs, STATA, SAS). [6]–[9] As methodological standards become more clear for conducting NMAs, guidelines should begin to consider how best to present and tailor NMAs to different end-users. Researchers themselves have identified areas of confusion related to NMAs and non-technical audiences are likely in need of greater support in understanding NMAs. Although NMAs build on many of the same concepts as traditional meta-analyses, these similarities are often not recognized and the same critiques that meta-analyses once faced (e.g. heterogeneity and combining studies) are frequently applied to NMAs. [10]

Although work is ongoing to develop standards for reporting NMAs and for critically appraising NMAs, [8], [11]–[12] consideration should also be given to identifying optimal presentation formats, i.e. determining not just ‘what’ to report but ‘how’ best to report it, and how to tailor information to different audiences who may be unfamiliar with NMAs. While good reporting practices are important to follow and contribute to clarity and transparency, they may be insufficient for good communication to non-technical audiences. Although reporting guidelines used by researchers enhance transparency, experience in the realm of clinical trials and evidence-based medicine has shown that they are insufficient for good communication to non-technical audiences and alternate tools and presentation formats such as decision-aids and clinical practice guidelines have been developed for patients and clinicians, respectively. While adequately reporting NMA details is a first essential step that provides a transparent description of the analysis and results to the reader, subsequently arranging this information in presentation formats that assist end-users in their understanding and/or application of the information is also an important consideration. Developing tools and alternate presentation formats that enhance the accessibility of NMAs may also be one approach to increasing their impact and value to clinicians and policymakers. While standards for transparent NMA reporting should be consistent, regardless of the audience or topic, different presentations formats may be appropriate for different audiences or topics. Some studies have focused on helping end-users such as clinicians interpret NMAs, [13]–[14] but it is unclear what guidance researchers have on how to optimally present NMAs to non-technical end-users such as policymakers.

A number of studies have surveyed current practices for presenting NMAs or explored different options for presenting NMAs [7], [15]–[17]. However, determining what guidance researchers are provided on how to present and tailor NMAs to different audiences and determining how it aligns with end-users needs is also an important piece of this puzzle. This can contribute to developing optimal presentation formats and knowledge translation approaches applicable to NMAs. [18] A systematic approach to knowledge translation could improve the accessibility of HTAs that are based on NMAs, thereby enhancing the legitimacy of and confidence in health policy decision-making processes. [1] Therefore, this study systematically reviewed current guidelines for conducting or reporting NMAs to determine what guidance researchers are provided on how to present NMAs. Our specific objectives were to: (1) determine if researchers are provided any guidance on how to present a NMA (2) determine if this guidance is targeted toward non-technical end-users such as policymakers or clinicians and (3) interpret these findings in the context of non-technical end-users' needs, who must apply the results of NMAs to policy or practice decisions.

## Methods

### Systematic Review

A systematic review was conducted following Cochrane methodology [19]. Guidelines for conducting or reporting on NMAs were included that primarily targeted statisticians, researchers and others who produce NMAs for the purpose of informing healthcare policy and practice decisions. In this review NMAs were defined as “an analysis that syntheses information over a network of comparisons to assess the comparative effects of more than two alternative interventions for the same condition; a network meta-analysis synthesizes direct and indirect evidence over the entire network, so that estimates of intervention effect are based on all available evidence for that comparisons.” (http://cmimg.cochrane.org/glossary/1#lettern). This definition encompasses other terminology that may be used including both “indirect comparisons” “mixed treatment comparisons” and “multiple treatment comparisons”. Formal NMA guidelines as well as interim guidance documents or working group documents that provided recommendations and were developed by collaborative groups or organizations with the intent of informing formal NMA guidelines were included. Studies were excluded if they were: editorials or opinion papers; original methodological articles on conducting NMAs; overviews or reviews of existing NMAs; guidelines not primarily focused on NMAs; or not the most recent or comprehensive versions of the guidelines. Guidelines on how to interpret or critically appraise NMAs were excluded because these are targeted primarily towards NMA audiences that must apply the results of NMAs and would not elicit information on what guidance researchers are provided on how to present NMAs to different end-users. Outcomes of interest were the type and frequency of information on presentation formats.

### Search Strategy

Databases searched included Medline (1996 to June 2014), EMBASE (1980 to June 2014) and the Cochrane Database of Systematic Reviews, using the earlier date limit of 2000 but no language restriction. The search concepts were ‘guidelines’, ‘indirect comparisons’, ‘network meta-analyses’ and ‘multiple or mixed treatment comparisons’. Grey literature was searched for unpublished reports using the CADTH Grey Matters checklist as a guide, in addition to other relevant resources [20]. Studies were also obtained through hand searching of selected journals and authors, reviewing reference lists of potentially relevant studies and suggestions from experts in NMAs.

### Study Selection

Citations were screened for relevance by one review author based on the title and abstract of identified articles. Two review authors independently reviewed the full text of potentially relevant guidelines to assess exclusion or inclusion.

### Critical Appraisal

Guideline quality was assessed using the AGREE II instrument [21]. AGREE II was designed, in part, to assess the quality of clinical practice guidelines. No instruments currently exist to assess methodological guidelines or guidelines for NMAs. Therefore, minor modifications to the AGREE II instrument were made for this study (see S1 Table for details).

### Data Extraction

One author extracted data, which was verified by a second author and disagreements were resolved by a third reviewer. The following data were extracted from guidelines (see S2 Table for detailed variable definitions).

1. *Guideline characteristics*: guideline purpose, guideline scope, general or disease-specific, target audience, year, geographic region, author affiliations and; providing a reporting template, sample tables, sample figures or a glossary.
2. *Presentation formats*: details on how to present data sources (evidence networks, individual trial characteristics, critical appraisal of individual trials), analysis methods (assumptions, heterogeneity and inconsistency, methodological concerns) and results (comparison with direct estimates, uncertainty, rankings, implications of findings). The target audience for different presentation formats was extracted when available.

Items related to presentation format were selected based on identification of key principles related to traditional meta-analyses and network meta-analyses [10] To be extracted, information was required on the format for presenting the information, not just that the information be provided or reported (i.e., focusing on ‘how’ to report not just ‘what’ to report).

Detailed guidance on how to conduct a NMA was not extracted (e.g. analytic approaches) and is not the focus of this systematic review.

### Data Analysis

The frequency of recommendations for presenting NMAs was tabulated and common trends assessed.

## Results

**Figure 1:**
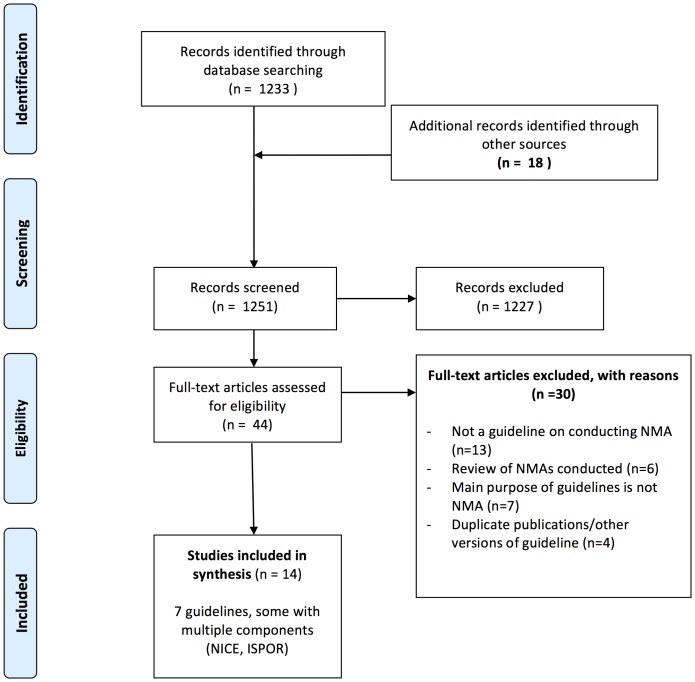
PRISMA Flow Diagram for Systematic Review of NMA Guidelines.

Of the 1251 citations identified, 14 reports, representing 7 guidelines, were included (see Fig. 1). [2], [22]–[34] Thirty reports were excluded, including a background document from the Cochrane Collaboration on the history of discussions within Cochrane on developing guidance for comparing multiple interventions in Cochrane Reviews. [35]

Key characteristics of the guidelines are outlined in Table 1 with the purpose of each guideline described in S3 Table.

**Table 1:** Characteristics of Network Meta-Analysis Guidelines.

Guideline	Geographic Region	Scope	Presentation Formats Recommended*	Sample Figures*	Sample Tables*	Reporting Template	Glossary or Definitions Provided	Acknowledging Non-Technical End-Users**
**ISPOR 2011**	International Collaboration	Reporting and Conducting	Yes	Yes	Yes	No	Yes	Yes
**CADTH 2009**	Canada	Conducting	No	Yes	No	No	No	Yes
**NICE DSU Series 2011**	UK	Reporting and Conducting	Yes	Yes	Yes	No	No	Yes
**PBAC 2008**	Australia	Conducting	Yes	No	No	No	Yes	Yes
**HAS 2009**	France	Conducting	No	Yes	Yes	No	No	No
**AHRQ 2010**	USA	Reporting and Conducting	Yes	No	No	No	No	Yes
**EUnetHTA 2013**	European Collaboration	Conducting	No	Yes	No	No	Yes	No

**Abbreviations**: AHRQ = Agency for Healthcare Research and Quality; CADTH = Canadian Agency for Drugs and Technologies in Health; EUnetHTA = European network for Health Technology Assessment; HAS = Haute Autorite de Santé; ISPOR = International Society for Pharmacoeconomics and Outcomes Research (ISPOR); NICE = National Institute for Health and Clinical Excellence; PBAC = Pharmaceutical Benefits Advisory Committee.

*Although actual recommendations on how to present NMAs were not provided in all guidelines, some example figures and tables were provided when illustrating how to conduct NMA, which could inform how to present NMAs.

**Although most guidelines acknowledged there were non-technical end-users of NMAs, only one (ISPOR) provided specific guidance on how to present information to them.

All guidelines provided guidance on how to conduct NMA and three (43%) also provided guidance on reporting NMA. [22]–[31] None of the guidelines were directed to any specific disease area or intervention and were generally applicable across all health technologies. Most guidelines (n = 5, 71%) were developed by HTA organizations around the world with the others developed by collaboratives with an interest in HTA, including the International Society For Pharmacoeconomics and Outcomes Research (ISPOR) and the European network for Health Technology Assessment (EUnetHTA). The target audience for all guidelines was researchers and decision-makers; two guidelines (29%) also identified health care professionals as part of the target audience. [29]–[31] Many guidelines (n = 5, 71%) specifically acknowledged how policymakers or other non-technical audiences use NMAs. However, only one guideline provided specific guidance regarding how to present NMAs to non-technical end-users [29]–[30]. For example, guidance suggested converting outcomes to measures policymakers might prefer such as relative risk, absolute risk reduction, number needed to treat. [29]–[30] None of the guidelines provided a full glossary of technical terminology used in NMAs, however three of the guidelines (43%) provided some definitions. [29]–[30], [32], [34]

The earliest guideline was published in 2008. [34] Most guidelines recognized the rapidly evolving field of NMA and noted that updates would be required and guidelines would be monitored for these changes. Guideline quality was generally low when critically appraised. Common limitations were related to narrow stakeholder involvement, lack of systematic development and few details related to implementation and applicability of guidelines. Policymakers frequently provided funding for the guidelines, however, authors' conflicts of interest were not reported in the majority of guidelines. Although recommendations were clearly identified in most guidelines, differences in terminology when comparing across guidelines may create confusion and lead to lack of clarity. More details on the critical appraisal are provided in S4 Table.

### Presentation Formats Identified in Guideline Recommendations, Sample Figures and Sample Tables

Of the seven guidelines that met the inclusion criteria, only four (57%) provided recommendations or guidance on presentation formats, as described in more detail in sections below. None provided an example template for how to report NMA. Although actual recommendations on how to present NMAs were not provided in all guidelines, some example figures (n = 5, 71%) and tables (n = 4, 57%) were provided when illustrating how to conduct NMAs. Sample figures and tables were generally related to presenting data sources (e.g. evidence network diagrams, trial characteristics tables) or results (e.g. forest plots, tables comparing direct and indirect estimates). These presentation formats are described in more detail below as they relate to each section.

The different areas of NMAs for which presentation formats were identified or recommended in guidelines and are outlined in Table 2.

**Table 2:** Network Meta-Analysis (NMA) Areas with Frequency of Presentation Formats Identified in Guidelines.

Areas of NMA	Recommendations on Presentation Formats	Formats Presented in Sample Figures or Tables
	n (%), N = 7	n (%), N = 7
**Included Data**		
Trial Network	3 (43)	3 (43)
Individual Trial Characteristics	4 (57)	4 (57)
Critical Appraisal	1 (14)	0
**Methods**		
Assumptions	2 (29)	1 (14)
Heterogeneity and/or Inconsistency	4 (57)	1 (14)
Methodological Concerns	2 (29)	0
**Results**		
Comparison of Direct and Indirect Effects	4 (57)	4 (57)
Uncertainty	3 (43)	4 (57)
Rankings	2 (29)	1 (14)
Implications of Findings	2 (29)	0

### Presenting Data Sources/Included Data

Three guidelines (43%) made recommendations on presenting evidence networks. [22]–[30], [34] All three recommended a graphical schematic of the evidence structure, which are often referred to as ‘network diagrams’. These diagrams outline relationships between the included studies and where direct and indirect evidence exists between therapies in the network. Diagram characteristics in the sample figures (n = 3, 43%) generally included features such as the use of solid lines for direct evidence and dashed lines for indirect evidence relationships; providing on the connecting line between two therapies the name, number of trials or direct results contributing to a comparison; arrowheads indicating which therapy is favoured in the comparison; and labeling or identifying the network geometry, e.g. star shapes, closed loops. However, specific recommendations on which features to include in the network diagram were not provided in any of the guidelines.

Two of the guidelines recommended flow diagrams outlining included and excluded studies and/or tables or lists identifying included and excluded studies. [22]–[30] When presenting lists or tables of included and excluded studies, clarifying which studies were identified in the systematic review versus which studies had sufficient information to be included in the network meta-analysis was requested in some guidelines. [22]–[30]

Four guidelines (57%) made recommendations on how to present details of individual trials. Two guidelines recommended presenting information in a table format [22]–[30] while the other two indicated it could be discussed in the text. [31], [34] Individual trial details included what treatments were compared, trial level data used in the analysis, trial level covariate values, or if individual participant data are available. [22]–[28] Other factors that could be effect modifiers were recommended for inclusion in tables such patient age, length of time with a disease, history of treatment or geographic region [29]–[30]. Two guidelines only made broad recommendations that sufficient study-level characteristics be provided and discussed. [31], [34] One guideline also specifically recommended presenting an assessment of individual trial quality as discussion in the text. [22]–[28]

When considering sample tables and figures related to data sources (n = 4, 57%), additional formats and characteristics were noted. For example, trial sponsor, population, therapies and doses, trial duration, size of treatment group, primary and secondary endpoints, blinding and study conclusions were included in one sample table [33] Three guidelines also recommended a table where each row is a study and each column is a treatment, with cells populated with absolute frequencies from the trials, which can clarify where direct evidence exists in the network while also demonstrating the raw data included in the analysis. [2], [22]–[30] In one guideline, additional columns reporting relevant trial characteristics were also added to the table. [22]–[30]

### Presenting Analysis Methods

Two guidelines (29%) recommended presenting information on general assumptions and on general methodological concerns as a description in the text. [22]–[30] When looking at more specific assumptions, four guidelines (57%) made recommendations on how to present issues related to heterogeneity and inconsistency. Multiple formats for presenting this information were identified including text descriptions, graphics and numerical estimates. For example, NICE technical support documents suggested graphics generated by ‘node splitting’, numerical estimates of the degree of heterogeneity, discussion of extent and sources of heterogeneity and plotting posterior mean deviances to identify inconsistencies. [22]–[28] Another guideline suggested presenting sensitivity analyses of including/excluding trials and providing a discussion of potential sources of heterogeneity. [34] Two other guidelines focused on presenting descriptions that included explicit statements and step-by-step description of analyses [29]–[30] or distinguishing between clinical, methodological and statistical heterogeneity. [31]

When considering sample formats in guidelines related to methodology (n = 1, 14%), various plots were included that would allow one to explore methodological assumptions when conducting an NMA such as leverage plots, density plots, interaction plots and residual deviance plots. [22]–[28] Sample table formats were also identified that included measures of model fit and heterogeneity, summary results for consistency and inconsistency models and that outlined results from different sensitivity analyses with and without covariate interactions.

### Presenting Results

Four guidelines (57%) made recommendations on how to present and compare direct estimates with indirect or mixed estimates. Both table formats and forest plots were recommended in two guidelines but there was no preference noted for one format over the other. [22]–[30] Generally, these formats were recommended so as to allow easy visual comparison of different results, e.g. outcomes by treatment arm, absolute and relative effect measures from trials, pairwise meta-analysis results and/or pooled NMA results. [22]–[30] Presenting results using a common reference standard was noted in two guidelines [22]–[30] and one guideline also recommended that all relevant pairwise comparisons should also be provided. [29]–[30] Two guidelines also recommended the use of multiple estimators to present results (e.g., relative risk, odds ratio, risk difference, number needed to treat). [29]–[30], [34] Tables providing relative treatment effects alongside estimates of heterogeneity were recommended in one guideline. [22]–[28] One guideline recommended a discussion of why the estimates differed but did not specify more details. [31]

Four guidelines (57%) provided sample tables that outlined direct, indirect and/or mixed estimates in table columns while each row represented a comparison of interest. [2], [22]–[30], [33] Other types of results were also compared in this format (e.g. analytic approaches such as Bayesian vs. frequentist or different sensitivity analyses). Two of these guidelines also provided samples of forest plots that included direct, indirect and mixed estimates and allowed for easy visual comparisons. [29]–[30], [33]

Three guidelines (43%) recommended presenting the uncertainty of estimates as credible intervals or confidence intervals around the point estimate, depending on the statistical analysis approach. [22]–[30], [34] In guidelines that provided sample figures or tables (n = 4, 57%), these intervals were usually provided in table columns or as a component of forest plots, along with the point estimate.

Two guidelines (29%) recommended presenting treatment rankings as either graphics or tables. [22]–[30] There was also considerable discussion in the guidelines on the challenges of presenting information on rankings so that it is not misinterpreted. For example, ensuring the information on the spread of rankings is provided [29]–[30] and that the probabilities of being best, second best, etc. are calculated was recommended in both guidelines. [22]–[30] One guideline specifically suggested presenting this information in the format of a rank-o-gram, which can incorporate multiple outcomes in one display. [22]–[28] Other possible formats published in peer-review literature were also recognized to be useful at times. [22]–[28]

Only one guideline provided a sample format of how to present rankings. [29]–[30] The guideline provided a table with each row being a different treatment versus the reference comparators and columns of results (ORs, % CrI) and columns of rankings and different probabilities of being best.

Two guidelines (29%) made recommendations on presenting the implications of findings as descriptions in the text. One guideline specifically referred to the implication of assumptions on results [22]–[28] The other identified implications with respect to validity of results; expectations compared with existing evidence, clinical rationale or biological rationale; relevance to real-world clinical and policy decisions; and the extent of possible bias and if it could lead to a different conclusion than a non-biased analysis. [29]–[30]

## Discussion

### Key Findings

Current NMA guidelines focus on how to conduct analyses but provide limited guidance to researchers on how to best present analyses. None of the guidelines provided reporting templates. Few provided specific advice on tailoring presentations to different end-users or extensive glossaries for non-technical readers, despite many guidelines being developed in HTA organizations for use, in part, by policymakers. Only four of the seven included guidelines provided advice on presentation formats or provided sample figures and tables to guide presentation of the NMA. This guidance focused primarily on presenting evidence networks, characteristics of individual trials, comparisons between direct and indirect estimates and assumptions of heterogeneity and/or inconsistency.

### Comparison with Other Literature and Policy Implications

Although reporting guidelines exist in other fields (e.g., economic evaluations, systematic reviews), due to the relatively innovative nature of NMAs, guidelines for the reporting and conducting of NMAs are less well-developed. Currently, NMA guidelines focus on defining standard methodological approaches and increasing the consistency and transparency of how NMAs are conducted. Corroborating this, two other reviews of NMA guidelines were identified but they compared methodological recommendations across guidelines and did not explore best practices of how to present NMAs to different audiences. [5], [36] The limited guidance may also be influenced by the evolving methodological approaches (e.g. Bayesian versus frequentist analyses) and various available software (e.g. WinBugs, STATA, SAS) that restrict how NMAs can be presented. Development of more universal and user-friendly software programmes could also contribute to assisting researchers in enhancing and standardizing how NMAs are presented for various end-users. However, with the growing use of NMAs and greater need for policymakers and other non-technical end-users to understand NMAs, determining how best to present NMAs has taken on greater prominence. [3]

The areas that were frequently identified in existing guidelines provide a starting point for focusing guidance for non-technical end-users, with interpretations offered in light of these end-users needs in using and understanding NMAs:

- ***Inclusion of trial details and network diagrams*** *.* This will also align with policymakers' need to understand the applicability of the analysis to their specific decision-making context. Providing graphical schematics (i.e., evidence network diagrams) may also help audiences better understand the relationships among the included studies. Looking at the shape of the network itself (e.g. star diagram, number of closed loops) can provide information on characteristics of the included evidence. [37], [38] In addition, there are many other styles of network diagrams that have been reported in literature and other trial characteristics that can be included (e.g. sample size, risk of bias). [11] Even though many guidelines provided examples of network diagrams, few guidelines made specific recommendations on their design or how to tailor them to policymakers and clinicians.
- ***Comparison between direct and indirect results.*** Questions often arise on why direct and indirect estimates may differ from each other and explanations could assist end-users. Although, NMA results may be complex, building on familiar approaches used to present traditional meta-analyses (e.g. forest plots) may provide end-users and researchers with greater comfort with and understanding of how to interpret NMA results. [10]
- ***Heterogeneity and inconsistency assumptions.*** Identifying presentation formats that provide audiences with clarity on the assumptions made in the analysis and help end-users understand their validity would be of value. Although assumptions other than heterogeneity and inconsistency are important in conducting NMAs (e.g. goodness of fit, random effects), different methods of presenting the validity of these other assumptions were not often noted.

Further developing guidelines for researchers on how to best present these aspects of NMAs to end-users of NMAs such as policymakers or others who want to enhance their understanding of NMAs should be considered in light of:

- ***Policymakers' preferences for evidence syntheses*** *.* Although policymakers may have limited experience with NMAs, they have often expressed views on how to present systematic reviews and traditional meta-analyses. Studies have reported that policymakers prefer presentation formats that can be quickly scanned, emphasize the bottom-line conclusions and clearly identify real-world implications [39]–[41]. Policymakers have also expressed a desire for understanding if the review is robust enough from a methodological perspective, to support decision-making. [12] Incorporating policymakers' preferences at this stage in the development of NMA guidelines would narrow the gap between policymakers and researchers when using and providing NMAs. This would also allow for the creation of NMAs that adequately balance both rigour and accessibility for those who must apply their results and understand their implications in the ‘real-world’. A recent ISPOR task force partially addresses this gap by developing a questionnaire for decision-makers to assess the relevance and credibility of NMAs. [12]
- ***Common principles related to the presentation of traditional meta-analyses*** *.* NMAs are based on many of the same principles as traditional meta-analyses. [10], [42]. Leveraging some of these same principles may be one approach that could help end-users better understand and appreciate NMAs. For example, conducting systematic searches for evidence, verifying homogeneity of populations when combining individual study results and presenting results with appropriate measures of precision or uncertainty are principles applicable to both traditional meta-analyses and NMAs.
- ***How researchers currently present NMAs*** *.* Current presentation of NMAs by researchers is extremely variable. This variability may be due, in part, to the range of current methodological approaches to conducting NMAs, software restrictions and the evolving methodology in the field of NMA. For example, Tan et al. (2013) reviewed 19 indirect treatment comparisons (IDCs) from NICE health technology assessments and found that researchers most frequently presented evidence network diagrams, model descriptions and tables and forest plots of results. [15] Based on this work approaches that may be most useful for non-technical audiences were identified and standardized graphical tools were developed that compare direct with indirect results and that summarize rankings. [16] An analysis from Chiamani et al. (2013) has also proposed new graphical options for presenting NMA methods and results that were developed using STATA. [7] For example, network diagrams that incorporate key trial characteristics and risk of bias assessments and contribution plots that emphasize how much direct versus indirect evidence is available in the analysis. Evaluating these different tools and formats to determine if they influence use or understanding of NMAs would be beneficial.
- ***Common limitations associated with conducting NMAs.*** Common limitations in NMAs may need to be presented very clearly for non-technical audiences. In a survey of 88 indirect comparisons, limitations included: an unclear understanding of underlying assumptions; inappropriate search for or selection of included trials; inappropriate or flawed methods; trial similarity not being objectively assessed; and an inappropriate combination of direct and indirect evidence. [43] A report on indirect comparisons submitted to NICE reported similar results, finding that common flaws were related to systematic review search methods, inappropriate pooling of heterogeneous data and suboptimal statistical methods. [44] More consistent reporting standards and optimized presentation formats in these areas may result in more clarity around expectations when conducting NMAs and ultimately enhance their quality.

Even with these considerations on how to optimize and tailor the presentation of NMAs to different end-users, the understanding and interpretation of NMAs may be challenging for non-technical end-users. The optimal presentation of NMAs should be considered as only one approach to build capacity in the field of NMAs and should be applied in concert with educational initiatives such as tutorials and workshops. For example, although few guidelines included glossaries of NMA terminology, glossaries or other tools may be available through other sources that are available to end-users (e.g., Cochrane Comparing Multiple Interventions Methods Group website, http://cmimg.cochrane.org).

### Strengths and Limitations

This is the first work to systematically review the guidance that researchers are provided on how to present NMAs and interpret these findings in the context of policymakers and other non-technical audiences. Identification of variables to extract was grounded in theoretical concepts of how NMAs relate to traditional meta-analyses. [10] Using this approach and building on traditional meta-analysis concepts could provide one avenue to facilitate understanding of NMAs among audiences familiar with traditional meta-analyses.

Methodological guidelines do not exist to critically appraise the quality of NMA guidelines and a tool for critically appraising clinical practice guidelines was adapted. Therefore, concepts unique to methodological guidelines may have been missed, however, the appraisal allowed identification of some key issues in the guidelines' development. For example, few guidelines reported authors' affiliations and/or conflicts of interest that may have influenced guideline recommendations. Conflict of interest may comprise not only financial interests but also intellectual conflicts such as guideline authors promoting methods with which they have the most experience. Given the small pool of experts in the emerging field of NMAs, it is challenging to determine the impact of intellectual conflict of interest; more time may be required before sufficient experience with different methods emerges and best practices in presenting NMAs can be identified that have widespread acceptability. Furthermore acceptability of guidelines by researchers and other end-users often depends on the level of stakeholder involvement in their development and consideration around how they will be implemented in practice. The quality of most guidelines was low around these aspects and should be considered in future if more efforts are devoted to enhancing guidelines on how to present NMAs for different stakeholders. Extracting qualitative data from various guideline documents requires judgment. The heterogeneity of guideline formats and objectives created challenges in data synthesis, however, common factors such as their development by HTA organizations and consideration of both researchers and policymakers suggests they have a similar intent. Also, the limited information available on presentation formats and broad data definitions applied in this review suggests that misclassification of data was unlikely to occur, e.g. it was unlikely that a figure presenting data sources would be missed or misclassified as a figure presenting results. A comprehensive search of the grey literature, electronic databases and other sources allowed for identification of a broad set of possible NMA guidelines. Although partial guidelines were excluded from this review, the novel but evolving nature of NMA guidance suggests they were not likely to yield additional data.

## Future Research and Conclusions

The focus on developing presentation formats should be in areas that were most frequently raised in current NMA guidelines and are important to researchers, including: trial details and evidence network diagrams; comparisons between direct and indirect results; and heterogeneity and inconsistency assumptions. However, limited guidance exists for researchers on how best to present NMAs in an accessible format for policymakers and requires further thought, and integration with policymakers needs. Knowledge translation approaches have frequently been applied to enhancing understandability and accessibility of clinical trial evidence for policy makers, healthcare providers and patients [45]–[47]. Tailored knowledge translation approaches have presented evidence syntheses in formats such as 1000 faces in decision aids and key messages in briefing notes tailored to policy makers [45]–[46]. Expansion of these approaches to NMAs has not yet occurred, but could be applied to develop technically accurate but simplified explanations of NMAs for policy makers or other non-technical audiences. For example, in addition to providing technical reports and scientific publications of NMAs, decision support tools could be developed. Tools could also be used to inform other educational initiatives or supplementary resources for policy makers and other general audiences to understand NMAs. Development of tailored presentation formats will allow policy makers to better apply the results of NMAs and enhance the transparency and legitimacy of HTA-informed decision-making processes.

## Supporting Information

S1 Table AGREE II for Critical Appraisal of Clinical Practice Guidelines.
(DOCX)

S2 Table Definitions of Variables and Data Extracted in Systematic Review.
(DOCX)

S3 Table Purpose of NMA Guideline Documents.
(DOCX)

S4 Table Quality of Network Meta-Analysis Guidelines as Assessed by modified AGREEII Instrument.
(DOCX)

S1 Checklist PRISMA Checklist.
(DOC)
