# Supplementary material for: What Guidance Are Researchers Given on How to Present Network Meta-Analyses to End-Users such as Policymakers and Clinicians? A Systematic Review
Source: PLoS One. 2014 Dec 17;9(12):e113277. doi: 10.1371/journal.pone.0113277 (PMC4269433; doi:10.1371/journal.pone.0113277)
Supplement: S1 Table — AGREE II for Critical Appraisal of Clinical Practice Guidelines. (DOCX) [file pone.0113277.s001.docx]

**Table S1. AGREE II for Critical Appraisal of Clinical Practice Guidelines ***

* To apply AGREE II (Brouwers et al., 2010) to the critical appraisal of methodological guidelines for network meta-analyses (rather than to clinical practice guidelines), the following minor modifications were made:

- For item 2 rather than ‘health’ question, the ‘methodological question(s) covered by the guideline is (are) specifically described.
- For item 11 rather than ‘health benefits, side effects and risks/, the ‘advantages and disadvantages have been considered in formulating the recommendations’
- Item 16 was removed: ‘the different options for management of the condition or health issue are clearly presented’

| **Domain** | **Item** |
| --- | --- |
| **Scope and purpose** | 1. The overall objective(s) of the guideline is (are) specifically described. |
|  | 1. The health question(s) covered by the guideline is (are) specifically described.* |
|  | 1. The population (patients, public, etc.) to whom the guideline is meant to apply is specifically described. |
| **Stakeholder involvement** | 1. The guideline development group includes individuals from all the relevant professional groups. |
|  | 1. The views and preferences of the target population (patients, public, etc.) have been sought. |
|  | 1. The target users of the guideline are clearly defined. |
| **Rigor of development** | 1. Systematic methods were used to search for evidence. |
|  | 1. The criteria for selecting the evidence are clearly described. |
|  | 1. The strengths and limitations of the body of evidence are clearly described. |
|  | 1. The methods for formulating the recommendations are clearly described. |
|  | 1. The health benefits, side effects and risks have been considered in formulating the recommendations.* |
|  | 1. There is an explicit link between the recommendations and the supporting evidence. |
|  | 1. The guideline has been externally reviewed by experts prior to its publication. |
|  | 1. A procedure for updating the guideline is provided. |
| **Clarity of presentation** | 1. The recommendations are specific and unambiguous. |
|  | 1. The different options for management of the condition or health issue are clearly presented.* |
|  | 1. Key recommendations are easily identifiable. |
| **Applicability** | 1. The guideline describes facilitators and barriers to its application. |
|  | 1. The guideline provides advice and/or tools on how the recommendations can be put into practice. |
|  | 1. The potential resource implications of applying the recommendations have been considered. |
|  | 1. The guideline presents monitoring and/ or auditing criteria. |
| **Editorial independence** | 22. The views of the funding body have not influenced the content of the guideline. |
|  | 23. Competing interests of guideline development group members have been recorded and addressed. |
