# Supplementary material for: What Guidance Are Researchers Given on How to Present Network Meta-Analyses to End-Users such as Policymakers and Clinicians? A Systematic Review
Source: PLoS One. 2014 Dec 17;9(12):e113277. doi: 10.1371/journal.pone.0113277 (PMC4269433; doi:10.1371/journal.pone.0113277)
Supplement: S2 Table — Definitions of Variables and Data Extracted in Systematic Review. (DOCX) [file pone.0113277.s002.docx]

**Table S2. Definitions of Variables and Data Extracted in Systematic Review**

| **Variable** | **Definition** |
| --- | --- |
| **Guideline Characteristics** | |
| Audience | Who is the intended audience for the guideline |
| Scope | Was the scope of the guideline related to conducting NMAs, reporting NMAs or both |
| Author Affiliations | Affiliations of guideline developers and authors including professional or academic collaborations, health technology assessment organizations, academic institutions, industry. |
| **Presentation Formats** | |
| *Data Sources/Included Data Formats* | |
| Trial Network | How to present the relationships between data points and structure of the evidence included in the NMA |
| Individual Trial Characteristics | How to present characteristics of the direct evidence the network is built upon |
| Risk of Bias | How to present methodological characteristics of the direct evidence that the network is built upon |
| *Analysis Methods Formats* | |
| Assumptions | How to present assumptions that are made and/or required for a NMA |
| Heterogeneity and Inconsistency | How to present potential sources of heterogeneity and/or inconsistency or any results evaluating these assumptions |
| Methodological Concerns | How to present any specific methodological issues that arise in an NMA |
| *Results Formats* | |
| Comparison of Direct and Indirect Effects | How to present comparisons of results that are obtained from direct evidence or from indirect and/or mixed evidence |
| Uncertainty | How to present issues around the uncertainty (or certainty) of NMA results |
| Rankings | How to present rankings in an understandable way to users, such that they are not misinterpreted |
| Implications of Findings | How to present the context surrounding NMA results, i.e. their impact and the implications of the finding |
