# Supplementary material for: What Guidance Are Researchers Given on How to Present Network Meta-Analyses to End-Users such as Policymakers and Clinicians? A Systematic Review
Source: PLoS One. 2014 Dec 17;9(12):e113277. doi: 10.1371/journal.pone.0113277 (PMC4269433; doi:10.1371/journal.pone.0113277)
Supplement: S3 Table — Purpose of NMA Guideline Documents. (DOCX) [file pone.0113277.s003.docx]

**Table S3. Purpose of NMA Guideline Documents**

| **Guideline** | **Purpose of Guidance** |
| --- | --- |
| **ISPOR 2011** | (1) Provides guidance on the interpretation of indirect treatment comparisons and NMA to assist policymakers and health-care professionals in using its findings for decision-making (2) Provides guidance on technical aspects of conducting network meta-analyses |
| **CADTH 2009** | To identify and review the different methods available for making indirect treatment comparisons; select methodological objectives using the Bucher indirect treatment comparison approach; to illustrate the application of various methods for indirect treatment comparisons |
| **NICE DSU Series 2011** | To support those presenting submissions to National Institute for Health and Clinical Excellence (NICE) by addressing the issues of the relative and absolute efficacy of interventions as it relates to evidence synthesis methods in decision-making |
| **PBAC 2008** | To provide a framework to help assess each indirect comparison to help judge the degree of confidence that can be given to relying on this assumption |
| **HAS 2009** | To provide a literature review to (1) present the background to the development of indirect comparison methods; (2) identify situations in which indirect comparisons can make a real contribution (3) present the various reliable indirect comparison methods (4) describe the main advantages and limitations of each methods (5) examine the validity of indirect comparisons (6) propose an evaluation and critical review method for indirect comparison studies |
| **AHRQ 2010** | To summarize publicly available guidance for, and current use of, meta-analytic methods for multiple treatment comparison evidence synthesis; to identify analyses using these methods and summarize their characteristics; to gain insight regarding the rationale for selection, implementation, and reporting of such methods from investigators |
| **EUnetHTA 2013** | To describe the main methods of direct, indirect and mixed treatment comparison available; to explain the main strengths and weaknesses of the methodologies; to discuss common issues that must be considered when interpreting results; to provide recommendations on the use of direct and indirect comparisons in a relative effectiveness assessment |
| **Abbreviations:** AHRQ=Agency for Healthcare Research and Quality; CADTH=Canadian Agency for Drugs and Technologies in Health; EUnetHTA=European network for Health Technology Assessment; HAS=Haute Autorite de Santé; ISPOR=International Society for Pharmacoeconomics and Outcomes Research (ISPOR); NICE=National Institute for Health and Clinical Excellence; PBAC=Pharmaceutical Benefits Advisory Committee | |
