# Supplementary material for: What Guidance Are Researchers Given on How to Present Network Meta-Analyses to End-Users such as Policymakers and Clinicians? A Systematic Review
Source: PLoS One. 2014 Dec 17;9(12):e113277. doi: 10.1371/journal.pone.0113277 (PMC4269433; doi:10.1371/journal.pone.0113277)
Supplement: S4 Table — Quality of Network Meta-Analysis Guidelines as Assessed by modified AGREEII Instrument. (DOCX) [file pone.0113277.s004.docx]

**Table S4. Quality of Network Meta-Analysis Guidelines as Assessed by modified AGREEII Instrument***

| **Guideline** | **Scope** | **Stakeholder Involvement** | **Rigour** | **Clarity** | **Applicability** | **Editorial Independence** | **Comments** |
| --- | --- | --- | --- | --- | --- | --- | --- |
| **ISPOR 2011** |  |  | x |  | x | x | **Strengths:** clear objective and audience; reviewed by experts before publishing; key stakeholders (researchers, industry, decision-makers) invited to join task force and could provide comments on drafts; reference list and citations throughout text; strengths and limits of evidence noted; includes accompanying tools; recommendations specific and clear; author affiliations reported  **Limits:** no systematic search for evidence; process for formulating recommendations unclear; pros and cons of recommendations not identified; no process for updating but areas for future research identified; implementation issues not discussed; no monitoring criteria; COI not reported; editorial independence from ISPOR unclear |
| **CADTH 2009** | x | x | x | x | x | x | **Strengths:** clear objectives; comprehensive literature search and selection of commonly reported methodologies; reference list and citations throughout text; strengths and limits of evidence noted; includes accompanying tools; COI reported; funded by decision-makers  **Limits:** audience not identified; reviewed by external academics/researchers before publishing but no other stakeholders involved (e.g. industry, decision-makers); recommendations not clearly identifiable and unambiguous (but providing recommendations was not a key objective); pros and cons of recommendations not discussed; process for formulating recommendations unclear; no process for updating but areas for future research identified; implementation issues not discussed; no monitoring criteria |
| **NICE DSU Series 2011** |  | x | x |  | x | x | **Strengths:** clear objective and audience; is regularly updated; reviewed by experts before publishing; reference list and citations throughout text; strengths and limits of evidence noted; recommendations specific and clear; includes accompanying tools; provides details on how to implement methods; author affiliations reported; editorial independence from funder (i.e. NICE)  **Limits:** no systematic search for evidence reported; involvement of broad stakeholder community unclear (e.g. decision-makers, industry); process for formulating recommendations unclear; pros and cons of recommendations not identified; no monitoring criteria; COI not reported |
| **PBAC 2008** |  | x | x |  | x | x | **Strengths:** clear objectives and audience; systematic search for evidence; reference list and citations throughout text; strengths and limits of evidence noted; recommendations specific and clear; developed and funded by decision-makers  **Limits:** stakeholder involvement or review of drafts outside of working group not reported (e.g. industry); criteria for selecting evidence unclear; process for formulating recommendations unclear; pros and cons of recommendations not identified; no process for updating or monitoring; no accompanying tools; implementation issues not discussed; COI not reported |
| **HAS 2009** | x | x | x |  | x | x | **Strengths:** clear objectives; systematic search for evidence conducted and selection criteria outlined; reference list and citations throughout text; strengths and limits of evidence noted; recommendations specific and clear; includes accompanying tools; developed and funded by decision-makers  **Limits:** audience unclear; stakeholder involvement or review of drafts not reported (e.g. industry); process for formulating recommendations unclear; pros and cons of recommendations not identified; no process for updating or monitoring; implementation issues not discussed; COI not reported |
| **AHRQ 2010** |  | x | x |  | x |  | **Strengths:** clear objectives and audience; reference list and citations throughout text; strengths and limits of evidence noted; recommendations specific and clear; to be updated as evidence evolves; COI reported; editorial independence from funder (i.e. AHRQ)  **Limits:** stakeholder involvement unclear; no systematic search for evidence reported; selection of evidence unclear; process for formulating recommendations unclear; pros and cons of recommendations not identified; no process for monitoring; no accompanying tools; implementation issues not discussed |
| **EUnetHTA 2013** |  | x | x |  | x | x | **Strengths:** objective and audience clearly defined; reviewed and validated by experts in HTA agencies who are target audience but other stakeholders not engaged; systematic search for evidence conducted and selection criteria outlined; recommendations are clear; reference list provided and cited within text; funded by decision-makers  **Limits:** process for formulating recommendations unclear; no process for updating or implementing recommendations; no accompanying tools; authorship and COI not reported |
| **Abbreviations:** AHRQ=Agency for Healthcare Research and Quality; CADTH=Canadian Agency for Drugs and Technologies in Health; EUnetHTA=European network for Health Technology Assessment; HAS=Haute Autorite de Santé; ISPOR=International Society for Pharmacoeconomics and Outcomes Research (ISPOR); NICE=National Institute for Health and Clinical Excellence; PBAC=Pharmaceutical Benefits Advisory Committee | | | | | | | |

‘x’ – indicates an issue was identified during critical appraisal of the guideline

* Minor modifications were made to the AGREEII instrument to assess methodological guidelines rather than clinical practice guidelines, as described in the Methods section of the Supplementary Materials.
